# A single-arm pilot of MyInspiration: a novel digital resource to support spiritual needs of patients undergoing cancer-directed surgery

**DOI:** 10.1007/s00520-024-08496-1

**Published:** 2024-04-16

**Authors:** Elizabeth Palmer Kelly, Maryanna Klatt, Jacqueline Caputo, Timothy M. Pawlik

**Affiliations:** 1 Department of Surgery, The Ohio State University Wexner Medical Center, James Comprehensive Cancer Center, 395 W 12th Ave. Suite 670, Columbus, OH USA; 2 Center for Integrative Health, Department of Family and Community Medicine, The Ohio State College of Medicine, Columbus, OH USA

**Keywords:** Spirituality, Cancer, Spiritual well-being, Surgery, Supportive care

## Abstract

**Purpose:**

This study aimed to assess the feasibility, acceptability, and satisfaction associated with the MyInspiration intervention, a digital spiritual support tool for patients undergoing cancer surgery. Additionally, we evaluated changes in spiritual well-being and the ability to find meaning in their experience with cancer before and after the intervention.

**Methods:**

This was a prospective, single-arm pilot study. Feasibility and acceptability were assessed by ratio of participants who completed all assessments among individuals who had signed consent forms. Satisfaction was assessed with 5 Likert-style questions around user experience. Patient spiritual well-being and finding meaning in their experience with cancer were measured at baseline and post-intervention.

**Results:**

Forty patients were enrolled, the majority of whom were female (80.0%) and diagnosed with breast cancer (52.5%), with an average age of 54.4 years (SD = 13.7, range 29.0–82.0). Regarding feasibility and acceptability, 76.9% of patients who consented to participate completed the full study protocol. In assessing satisfaction, 59% of patients were satisfied with the overall experience of MyInspiration. There was no difference in spiritual well-being pre-/post-intervention. There was a difference in pre (*M* = 1.95, SD = .95) and post (*M* = 2.23, SD = .86) scores relative to “finding meaning in the cancer experience” with a mean difference of 0.28 (*p* = 0.008).

**Conclusion:**

MyInspiration was feasible and acceptable to patients, and the majority were satisfied with the tool. The intervention was associated with changes in patients’ ability to find meaning within their cancer experience. A randomized control trial is needed to evaluate the efficacy of the tool in a broader population of patients with cancer.

## Introduction

Cancer is a life-threatening diagnosis that can induce existential distress among patients who face multiple uncertainties about a new diagnosis and treatment plan. The spiritual (and religious) beliefs of cancer patients exert direct and indirect influences on their care experiences, treatment decisions, and clinical outcomes [1, 2]. In the context of healthcare, spirituality is defined as “the aspect of humanity that refers to the way individuals seek and express meaning and purpose and the way they experience their connectedness to the moment, to self, to others, to nature, and to the significant or sacred” [3]. Patients often perceive spirituality as a source of self-reflection, encouragement, and motivation with many individuals considering spirituality/religion as a source of “inspiration” [4]. Surgical intervention for a malignancy can be particularly challenging and demanding as there are often physical, psychological, and emotional burdens. The role of spirituality and spiritual well-being as a source for patient coping and addressing existential distress is frequently overlooked, however, in the context of cancer surgery.

Recent research has underscored the significance of spirituality as a valuable interpersonal and intrapersonal resource for cancer patients and their family members. In particular, spirituality can instill hope, imbue meaning into the cancer experience, enhance psychological adjustment to cancer-related challenges, and improve overall quality of life [5–9]. Additionally, spirituality can influence patient treatment-related decisions, including engaging in complementary therapies, enrolling in clinical trials, and transitioning to palliative and hospice care [10–13]. Several barriers may impede the provision of spiritual care within healthcare settings, including lack of spiritual care experts on staff (e.g., pastoral care), time limitations, and provider level of comfort [14–16]. In turn, many individuals undergoing and recovering from cancer surgery may benefit from non-medical resources to aid in their coping process.

To date, no prospective trials of interventions supporting patient spirituality in the context of cancer surgery have been conducted. To address this issue, our long-term research goal has been to develop a digital resource to support patient spirituality that can be customized based on patient preferences and administered to cancer patients and their family members before, during, and after cancer treatment. Using a single-arm pilot study design, we hypothesized that the MyInspiration intervention would feasible, acceptable, and associated with patient satisfaction. Additionally, we aimed to evaluate changes in patient ability to find meaning in their experience with cancer and spiritual well-being before and after the intervention.

## Methods

### Study design, participants, and procedure

**Fig. 1:**
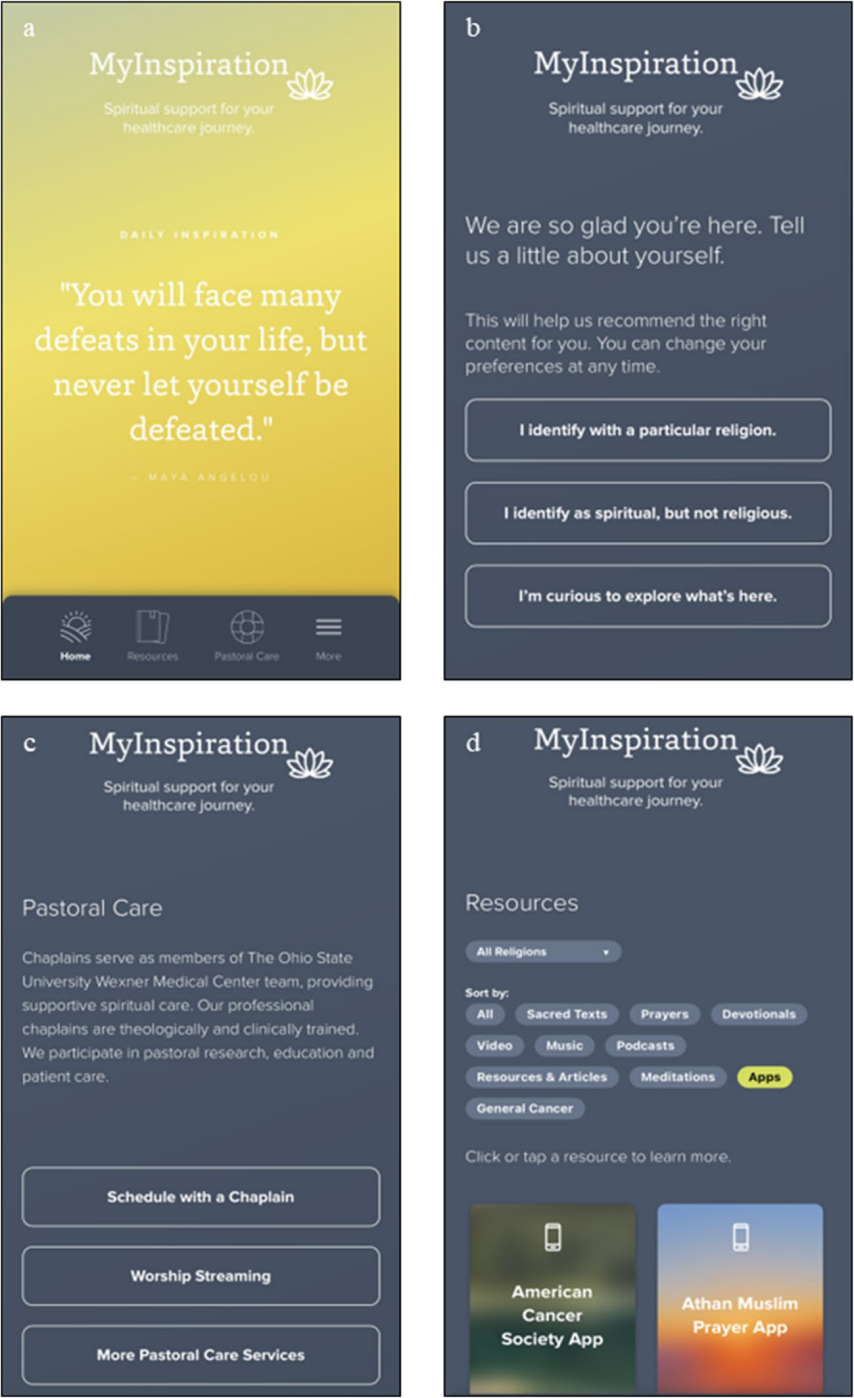
**a**–**d** MyInspiration digital tool

This study was a single-arm, prospective pilot trial of MyInspiration. In its current form, MyInspiration is a web-based digital tool designed to address patient spiritual needs by delivering content tailored to individual spiritual orientation. The current version of MyInspiration has patient resources across five domains: (1) written (e.g., sacred texts), (2) audio/visual (e.g., music/podcasts), (3) physical (e.g., submit requests for religious tools and foods), (4) communal (e.g., group worship), and (5) relational (e.g., request a chaplain or religious leader). MyInspiration offers a wide array of resources, some of which are general in nature (e.g., guided imagery, mindfulness), while others are customized based on the patient’s spiritual identity and specific needs. For instance, patients who identify as Hindu can indicate their religious preference during the onboarding or orientation process. Subsequently, if the patient expresses a desire for a religious text, the individual will receive the Vedas and the Upanishads—specific to their faith—rather than an exhaustive list of all religious texts. Additionally, every patient has access to the general resources available to all patients. Patients who do not specify a religious preference can explore all the resources offered by MyInspiration (Fig. 1).

Patients at The Ohio State University Comprehensive Cancer Center (OSUCCC) who were scheduled for future surgery as part of their treatment were recruited from August 1 through December 31, 2022. Eligible participants needed to be over 18 years of age and proficient in English. All recruited patients had access to a personal smart device to complete study activities. Recruitment for potential participants was carried out through three primary channels: (1) flyers, (2) MyChart, and (3) phone. Flyers were distributed to surgical oncology clinics to display for patients. The recruitment flyer contained information about the study and contact information for a member of the research team to receive additional information regarding the study. For recruitment through MyChart, honest brokers were utilized. Honest brokers are individuals who are not involved with the research study team; these “brokers” query patients who meet study eligibility and send individuals an approved recruitment email via MyChart inbox. Investigators only received patient information if the patient indicated interest in the study. Another method of recruitment involved research associates who pre-screened potential patients scheduled for appointments in the surgical oncology clinics prior to an appointment. If the patient appeared eligible, research staff members asked for physician approval to contact the patient. If the physician approved, research staff called the patient prior to the appointment and introduced the study.

For all three recruitment methods, participants who expressed interest to participate were contacted by research staff, screened for eligibility, and given the opportunity to ask any questions. If participants met eligibility criteria, they were provided with a consent form online via RedCap. After obtaining consent, patients received instructions to guide them through the process of accessing and utilizing MyInspiration, as well as completing all baseline measures. Participants were invited to take part in the study for a period of 30 days, which included any inpatient stays, if applicable. Efforts were made to schedule the surgical procedure in such a way that it fell within the middle of the study window, allowing patients to use MyInspiration both leading up to and following their surgical procedure. Upon successful completion of the 30-day follow-up survey, participants received online Amazon gift cards valued at $20 as compensation for their participation. This study protocol received approval from the Institutional Review Board at The Ohio State University (Protocol #2021C0010).

### Measures

Individual patient (i.e., gender, relationship status, race, age) and disease-level (i.e., cancer type, stage, time since diagnosis) demographics were extracted from the electronic medical record at the conclusion of study activities. Feasibility and acceptability were assessed through recruitment and retention metrics (ratio of patients that consented and completed all assessments). Reasons why participants declined to enroll were also ascertained. Satisfaction was assessed with 5 Likert-style questions assessing patients’ user-experience with MyInspiration.

Patient spiritual well-being and finding meaning in their experience with cancer were measured at baseline and post-intervention. Spiritual well-being was assessed with the Functional Assessment of Chronic Illness Therapy-Spiritual Well-Being 12 item scale (FACIT-SP-12) [17]. The FACIT-SP-12 is the most frequently used measure of spiritual well-being amongst people with cancer and is part of the FACIT measurement system. Internal consistency was measured using Cronbach’s alpha and was deemed acceptable (> 0.70) for all subscales of the FACIT-Sp-12: *α* = 0.87 for faith (4-items), *α* = 0.88 for combined meaning/peace (8-items), *α* = 0.78 for meaning alone (4-items), *α* = 0.83 for peace alone (4-items), and *α* = 0.89 for Total FACIT-Sp-12 (12-items) [18, 19]. Responses on the FACIT-Sp-12 range from 0 (“not at all”) to 4 (“very much”). Subscale scores range from 0 to 16, while total scale score ranges from 0 to 48, with higher scores indicating better spiritual well-being [20]. The Impact of Cancer Scale (IOC) was developed to assess the influence of cancer on quality of life. The IOC has eight subscales. For the current study, “meaning of cancer” subscale was utilized, which examines personal growth related to the cancer experience (e.g., having had cancer turned into a reason to make changes in my life). The Likert-style response scale was the same as the FACIT-Sp-12. Subscale scores were averaged, with a possible range from 0 to 4 with higher scores being more positive. The IOC has published psychometric properties [21].

### Data analysis

Descriptive statistics were used and presented as frequency (relative frequency: %) and mean (*M*, standard deviation: SD, range) for categorical and continuous data, respectively. It was determined prior to study activities that feasibility/acceptability > 75% was deemed acceptable. Paired *t*-tests were used to examine differences scores on baseline and exit assessments. All analyses were performed using SPSS v28 with significance established at *α* < 0.05.

## Results

### Enrollment and demographics

**Fig. 2:**
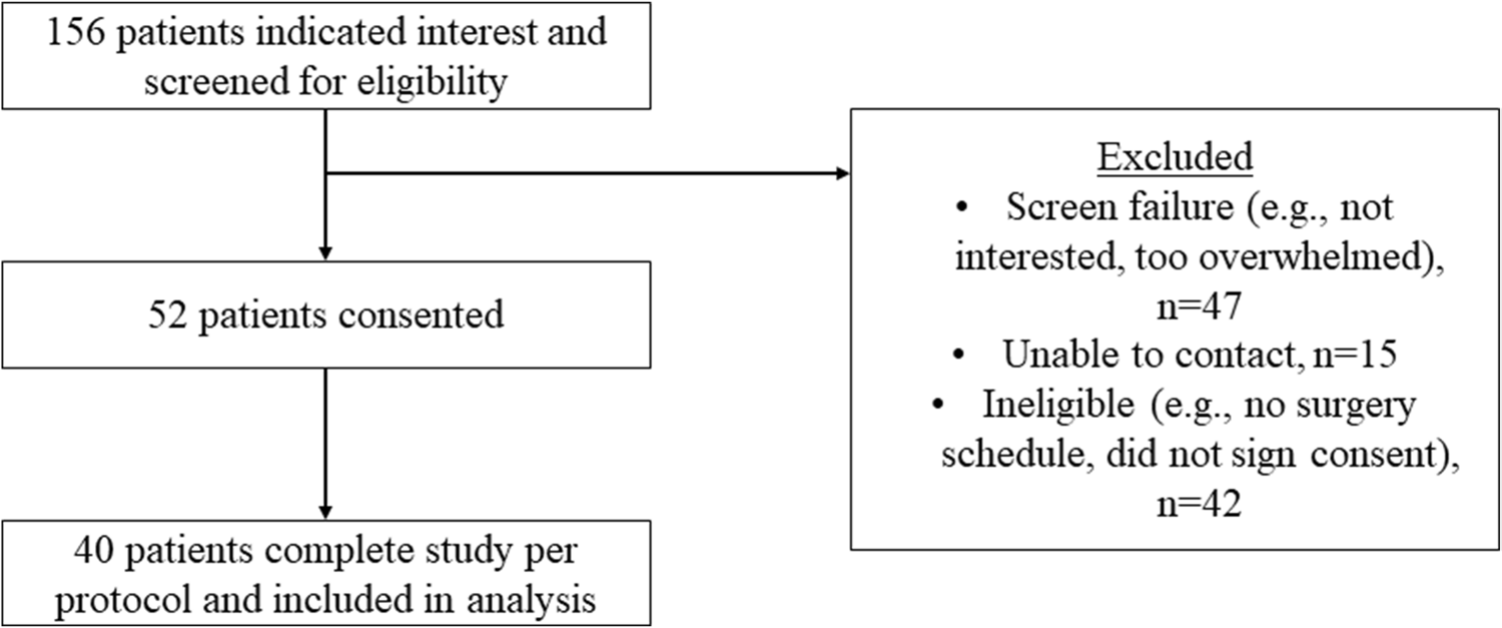
Patient enrollment flow chart

Overall, 156 patients indicated interest in the study. Among these patients, 104 patients were excluded because of screen failure (*n* = 47), unable to be contacted (*n* = 15), or deemed ineligible (*n* = 42), resulting in 52 patients who signed consent forms to participate. Among these 52 patients, 40 completed the study per protocol and were included in the final analytic sample (Fig. 2). The majority of patients were female (*n* = 32, 80.0%), married (*n* = 25, 62.5%), and White (*n* = 38, 95.0%) with an average age of 54.4 years (SD = 13.7, range 29.0–82.0). The most common cancer type was breast (*n* = 21, 52.5%) and most patients had early-stage disease (i.e., stage 0–II; *n* = 24, 64.9%). The average time from the time of diagnosis to survey assessment relative to the MyInspiration intervention was 274.3 days (SD = 338.49, range 37.0–1714.0) (Table 1).

**Table 1:** MyInspiration pilot sample demographics

	*M*(SD)	Range
Age	54.35 (13.69)	29.0–82.0
Time since diagnosis (days)	274.30 (338.49)	37.0–1714.0
		*N *(Valid %)
Gender	Male	8 (20.0)
	Female	32 (80.0)
Relationship status	Married	25 (62.5)
	Not married	15 (37.5)
Race	White	38 (95.0)
	Black/African American	2 (5.0)
Cancer type	Breast	21 (52.5)
	Not breast	19 (47.5)
Stage	0–2	24 (64.9)
	3–4	13 (35.1)

### Feasibility, acceptability, and satisfaction

**Fig. 3:**
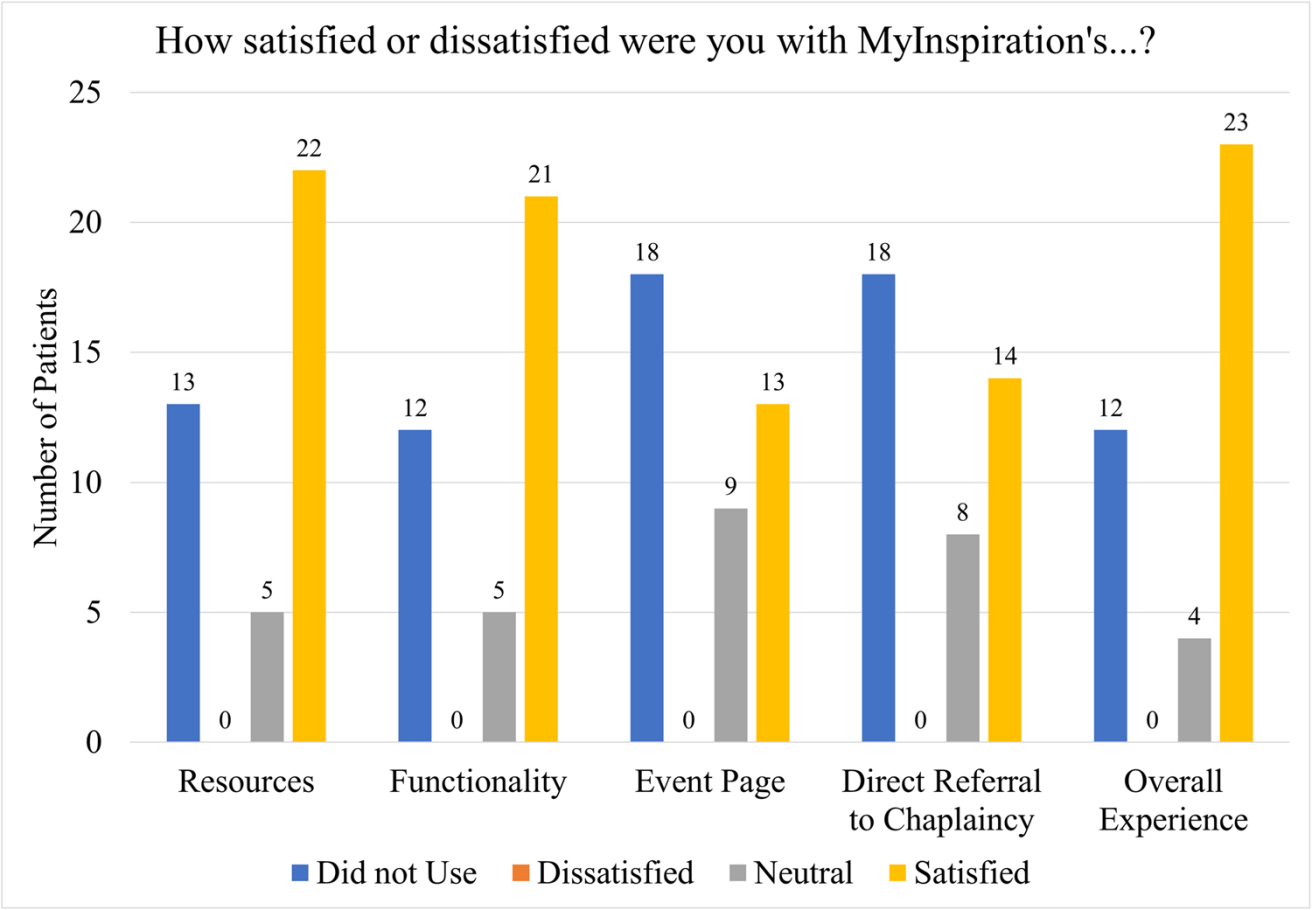
Patient satisfaction ratings. Not all numbers total 40 due to lack of participant response to some questions

Regarding feasibility and acceptability, 76.9% of patients who consented to participate completed the full study protocol. In assessing patient experience with MyInspiration, 59% of patients (*n* = 23) were satisfied, while other patients were either neutral (*n* = 4, 10.3%) or indicated they did not use the resource (*n* = 12, 30.9%); no patient indicated they were dissatisfied with their overall experience with MyInspiration. Similarly, 55.3% of patients indicated they were satisfied with the functionality of MyInspiration (*n* = 21), while 13.2% were neutral (*n* = 5). Regarding the specific components of MyInspiration (i.e., Resources, Event Page, Direct Referral to Chaplaincy), patients were most satisfied with the available resources (*n* = 22, 55.0%), while 18 patients indicated that they did not use the event page or direct referral to chaplaincy (45.0%). Of note, no patients indicated they were dissatisfied with MyInspiration’s resources, functionality, event page, and direct referral to chaplaincy (Fig. 3).

### Differences in spiritual well-being and meaning of cancer

There were no differences noted among scores for baseline (*M* = 35.45, SD = 7.23) and exit assessments (*M* = 35.83, SD = 7.65) for spiritual well-being, as assessed by the FACIT-Sp-12 total score (*p* = 0.652) or relative to the meaning, peace, and faith subscales (all *p* > 0.05). There was, however, a difference in baseline (*M* = 1.95, SD = 0.95) and exit (*M* = 2.23, SD = 0.86) scores for the meaning of cancer subscale (mean difference, 0.28; *p* = 0.008) (Table 2).

**Table 2:** Differences in spiritual well-being and meaning of cancer

	*M* (SD)	Range	Mean difference	*p*
FACIT total baseline	35.45 (7.23)	14.0–47.0	.38	.652
FACIT total exit	35.83 (7.65)	12.0–46.0		
FACIT meaning baseline	13.60 (2.36)	3.0–16.0	-.25	.333
FACIT meaning exit	13.35 (2.76)	2.0–16.0		
FACIT peace baseline	11.18 (2.57)	5.0–16.0	.23	.524
FACIT peace exit	11.41 (2.53)	6.0–15.0		
FACIT faith baseline	10.73 (4.28)	0.0–16.0	.63	.170
FACIT faith exit	11.35 (4.22)	0.0–16.0		
Meaning of cancer baseline	1.95 (.95)	0.0–4.0	.28	**.008**
Meaning of cancer exit	2.23 (.86)	0.0–4.0		

Bold value indicates *p* < 0.05

## Discussion

Healthcare teams are entrusted with the responsibility to deliver high-quality, patient-centered care to support cancer survivors throughout their entire cancer journey. This comprehensive approach should encompass medical aspects of care, but also the patient perspective around non-medical, psycho-social, and spiritual support [22, 23]. Surgery, which is often a component of cancer care, carries both medical and psychosocial-spiritual implications. While patients, caregivers, and family members often express a desire to discuss their spiritual needs with healthcare providers, this facet of a patient’s life is frequently overlooked within the healthcare setting by healthcare professionals [1, 24, 25]. The present study is significant as it represents the first prospective pilot of an intervention designed to bolster patient spirituality within the context of cancer surgery. The results were promising as the MyInspiration tool demonstrated both feasibility and acceptability among patients. Furthermore, a majority expressed satisfaction with their experience using the tool. Additionally, evidence suggested an improvement in a patients’ ability to find meaning in their cancer journey with assistance of the MyInspiration tool.

One notable aspect of the current study was the high level of satisfaction reported by patients regarding their overall experience with MyInspiration. Of note, no patient expressed dissatisfaction with the tool’s functionality or its specific components. Nevertheless, it is noteworthy that some patients did not choose to utilize the MyInspiration tool, suggesting an opportunity to enhance its functionality and redesign to appeal to a broader range of users. One avenue for improvement could involve the addition of new components to the tool. For instance, given the established relationship between spirituality and treatment decisions, particularly concerning end-of-life choices [12, 20, 21], the incorporation of information or an intervention to support advanced care planning within MyInspiration may have relevance for some patients who are anticipating surgery for a malignant indication. Indeed, a previous study by our team noted an association between patient spirituality, religion, and the presence of an advance care plan among cancer patients [22]. Furthermore, the MyInspiration tool could place greater emphasis on inclusive, spirituality-based interventions such as mindfulness and/or meditation. The inclusion of mindfulness exercises could be pertinent to engage patients who do not consider themselves to be spiritual yet find the more general concept of mindfulness helpful. One strength of the MyInspiration tool is that it leverages mobile technology, which has shown promise in reaching underserved populations to deliver interventions aimed at enhancing quality of life and overall well-being. In addition, the use of religion and spirituality may be critical in outreach to traditionally under-represented populations, who traditionally are more inclined to religion and spirituality [23, 24].

Improvements in patients’ capacity to derive meaning from their cancer experience were observed when comparing baseline and exit assessments. While the interpretational scope may be limited by the small-scale nature of this pilot study, there was a difference in baseline and exit scores for the meaning of cancer subscale (Table 2). “Meaning making” holds a substantial existential relevance and serves as a prevalent coping mechanism for individuals with cancer [20, 21]. The act of discovering meaning within the cancer experience is linked with several favorable outcomes, including improved psychological well-being, enhanced social adjustment, reduced overall distress, and the reestablishment of a profound sense of purpose and personal values [20–22]. In this context, spirituality and spiritual well-being may be pivotal resources for many patients navigating the challenges of cancer surgery. Consequently, it is critical for healthcare providers to gain a comprehensive understanding of the specific resources that hold significance for their patients during the perioperative phase, including spirituality. To this end, the MyInspiration tool may be a potential easy-to-use means to assist patients in finding meaning — spiritual and otherwise — as they go through their cancer journey.

There were several limitations related to the current study. As a pilot study, the sample size was small and there was no control arm. Given the limited sample size in this pilot study, the population was relatively homogeneous relative to gender, cancer type, and race/ethnicity. A comprehensive randomized controlled study is warranted and being developed to establish the efficacy of MyInspiration in a larger cohort of patients.

In conclusion, this pilot study demonstrated the feasibility and impact that a digital tool to address spiritual needs among cancer surgery patients. The MyInspiration tool had good functionality and patient satisfaction with the various components of the tool was high, with no reported dissatisfaction. In addition, the MyInspiration tool was associated with a difference in helping find “meaning” during their cancer surgery journey. Lessons learned from this pilot study will be applied to a future full-scale, randomized controlled efficacy trial using MyInspiration. Embracing the spiritual dimension of patient care is critical to improve patient quality of life, well-being, as well as patient-centered care for individuals navigating the cancer care trajectory.

## Declarations

This study was performed in line with the principles of the Declaration of Helsinki. Approval was granted by the Ethics Committee of The Ohio State University (Protocol #2021C0010). Informed consent was obtained from all individual participants included in the study.
